# TREM2 and microglial immunity in Alzheimer’s disease: mechanisms, genetics, and therapeutic opportunities

**DOI:** 10.3389/fimmu.2026.1739875

**Published:** 2026-02-18

**Authors:** Tianqing Wang, Xinru Liu, Xifeng Wang, Fuzhou Hua, Lei Yan

**Affiliations:** 1 The First Clinical Medical College, Nanchang University, Nanchang, Jiangxi, China; 2 Department of Anesthesia, People’s Hospital of Xinjiang Uygur Autonomous Region, Urumqi, China; 3 Graduate School of Xinjiang Medical University, Urumqi, China; 4 Department of Anesthesiology, The Second Affiliated Hospital, Jiangxi Medical College, Nanchang University, Nanchang, Jiangxi, China

**Keywords:** Alzheimer’s disease, immunotherapy, microglia, neuroimmunology, TREM2

## Abstract

Alzheimer’s disease (AD) is increasingly recognized as a disorder of innate immune dysregulation within the central nervous system. The triggering receptor expressed on myeloid cells 2 (TREM2), a microglial immunoreceptor, has emerged as a pivotal genetic risk factor for late-onset AD, underscoring the critical role of neuroimmune interactions in disease pathogenesis. This review synthesizes recent advances concerning TREM2’s modulation of core microglial functions, including phagocytosis, inflammatory signaling, cellular metabolism, and survival, processes that are essential for responding to amyloid-β plaques and neuronal damage. We highlight the TREM2-APOE pathway as a central mechanism driving the disease-associated microglia (DAM) phenotype and examine how loss-of-function mutations such as *R47H* disrupt immune surveillance, aggravate amyloid pathology, and promote neuroinflammation. Additionally, we explore the diagnostic and therapeutic potential of soluble TREM2 (sTREM2) and TREM2-targeted immunotherapies, which enhance plaque encapsulation and cognitive outcomes in preclinical models. By integrating genetic, molecular, and clinical evidence, this review establishes TREM2 as a keystone regulator linking amyloidosis, tauopathy, and neuroinflammation, highlighting its promise as a target for disease-modifying therapies.

## Introduction

AD represents a progressive neurodegenerative disorder with a multifaceted genetic and pathological background. It currently affects more than 44 million individuals globally and remains the most common cause of dementia in the aging population (1). Neuropathologically, AD is defined by the accumulation of extracellular amyloid-β (Aβ) plaques and intracellular neurofibrillary tangles composed of hyperphosphorylated tau. These hallmark lesions are closely associated with synaptic failure, chronic neuroinflammation, and extensive neuronal loss. A central event in AD pathogenesis is the dysregulated homeostasis of Aβ, stemming from an imbalance between its production and clearance, leading to widespread peptide deposition both within and outside neurons.

**Figure 1:**
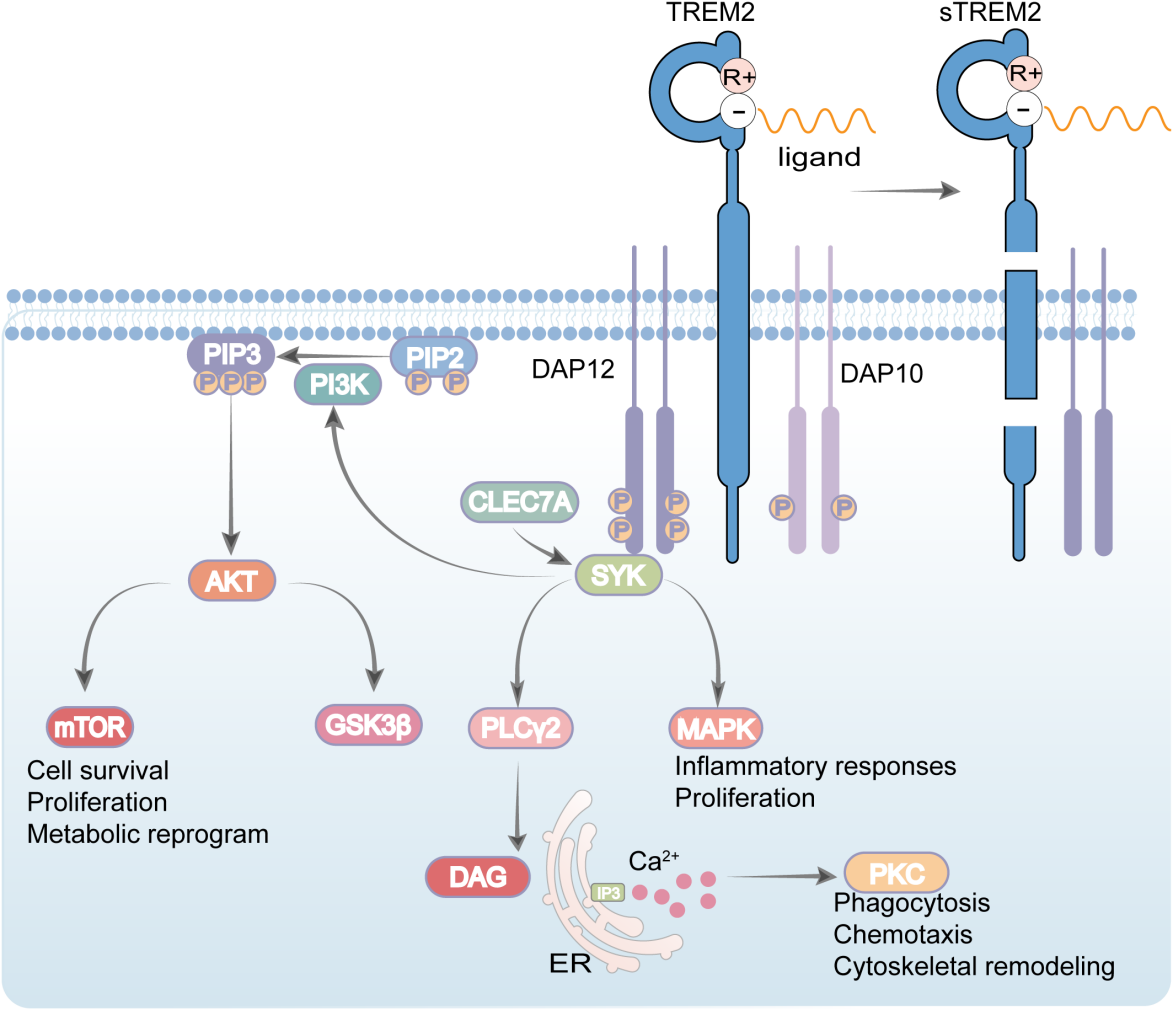
Schematic illustration of TREM2 signaling. Upon ligand binding, the Triggering Receptor Expressed on Myeloid cells 2 (TREM2) associates with the adaptor DAP12 to initiate intracellular signaling. Key downstream pathways include the PI3K–AKT–mTOR axis, which supports cell survival, proliferation, and metabolic reprogramming, and the PLCγ–mediated generation of DAG and IP_3_, leading to PKC activation and calcium flux that regulate phagocytosis, chemotaxis, and cytoskeletal reorganization. Signaling through CBL, GSK3β, and MAPK pathways further modulates inflammatory responses and proliferation. Collectively, this integrated network highlights the essential role of TREM2 in linking immune sensing to fundamental cellular functions, with particular relevance to microglial activity and neuroinflammatory regulation.

Beyond classical neuropathological features, dysregulated immune mechanisms are now recognized as critical contributors to disease progression. These mechanisms particularly involve microglia, the resident innate immune cells of the central nervous system (CNS). TREM2 is a microglial immunoreceptor deeply implicated in innate immune responses and neuroinflammatory processes (2). Structurally, TREM2 is a transmembrane protein of the immunoglobulin superfamily, consisting of an extracellular domain, a single-pass transmembrane helix, and a short cytoplasmic tail. It is highly expressed in microglia throughout key brain regions such as the basal ganglia, corpus callosum, and brainstem (3). Functionally, TREM2 serves as a pivotal regulator of microglial activity, modulating phagocytosis of apoptotic debris, damaged myelin, and Aβ aggregates (4, 5). Upon engagement with ligands such as apolipoproteins and phospholipids, TREM2 associates with the adaptor protein DNAX activating protein of 12 kDa (DAP12) to initiate intracellular signaling cascades that promote microglial survival, proliferation, metabolic reprogramming, and chemotaxis (6, 7). Central to this signaling is the phosphorylation of spleen tyrosine kinase (SYK) and subsequent activation of downstream effectors including phospholipase C gamma (PLCγ), phosphatidylinositol 3-kinase (PI3K), and mitogen-activated protein kinase (MAPK) pathways, which collectively orchestrate functional responses such as cytokine release, synaptic pruning, and autophagy (8), as illustrated in **Figure 1**. In recent years, genome wide association studies (GWAS) have substantially expanded our understanding of genetic risk factors underpinning AD, notably identifying triggering receptor expressed on myeloid cells 2 (*TREM2*) as one of the most significant susceptibility genes after *Apolipoprotein E (APOE)*, *Amyloid Precursor Protein (APP)*, and *Presenilin 1 (PSEN1)* (9, 10).

Notably, rare variants in *TREM2* (e. g. *R47H*) significantly elevate AD risk by impairing microglial function and immune surveillance, underscoring its role in maintaining CNS immune homeostasis (11, 12). In the context of AD, microglia transition to a disease-associated (DAM) phenotype, characterized by altered transcriptional profiles and enhanced phagocytic capability, yet also by potential loss of homeostatic functions and exacerbated neuroinflammation (13, 14). Through crosstalk with complement and chemokine systems such as CX3CL1/CX3CR1, TREM2 also influences synaptic plasticity and neuronal integrity (15, 16).

Given its central role in regulating neuroimmune interactions, TREM2 has emerged as a compelling therapeutic target for modulating innate immunity in AD. This review synthesizes recent advances to delineate the multifaceted role of TREM2 at the nexus of neuroimmunology and AD pathogenesis. Its innovative perspective lies in integrating evidence across genetic, molecular, and clinical domains to propose a unified framework of TREM2 function. We particularly highlight several emerging and integrative concepts: First, the central role of the TREM2-APOE axis in driving the disease-associated microglia (DAM) phenotype and its implications for lipid metabolism and immune response crosstalk. Second, the dual and stage-dependent impact of TREM2, where its deficiency can attenuate plaque load early but exacerbate pathology and neuritic dystrophy at advanced stages, underscoring the critical importance of timing in therapeutic intervention. Third, the extension of TREM2’s influence beyond amyloid pathology to include the modulation of tau hyperphosphorylation and spreading, potentially via the PI3K/Akt/GSK-3β pathway. Fourth, the recognition of systemic and metabolic dimensions, as TREM2 dysregulation is evident peripherally and linked to metabolic comorbidities of AD. Finally, we explore the translational frontier, evaluating novel immunotherapeutic strategies such as TREM2-agonizing antibodies (e.g., AL002c, ATV: TREM2) and biomarker potential of soluble TREM2 (sTREM2), which collectively represent a paradigm shift towards immune-targeted, disease-modifying therapies. By bridging these mechanistic insights, this review aims to articulate the promise of TREM2 as a keystone regulator linking amyloidosis, tauopathy, and neuroinflammation, thereby illuminating new paths for therapeutic discovery.

## TREM2 in neuroimmune dysfunction and AD pathogenesis

TREM2 has emerged as a critical immunomodulatory receptor in AD, primarily governing microglial responses to neuropathological changes. Under physiological conditions, TREM2 supports microglial homeostatic functions through its interaction with the DAP12-SYK signaling axis, which maintains cellular metabolic fitness through regulation of mechanistic target of rapamycin (mTOR) activation, protein synthesis, and energy metabolism (8, 17). This signaling pathway enables microglia to acquire a protective transcriptional signature essential for their normal surveillance functions (13, 18). Beyond metabolic support, TREM2 regulates microglial communication with other neural cells, particularly through the secretion of factors that modulate astrocytic synaptic phagocytosis, thereby contributing to synaptic integrity maintenance (19, 20).

In the Alzheimer’s disease brain, TREM2 demonstrates complex region-specific alterations that reflect its selective involvement in disease processes. Cortical TREM2 levels correlate positively with clinical AD diagnosis, cognitive impairment, and amyloid-β load, while caudate TREM2 expression primarily associates with local microglial activation rather than conventional AD markers (21). With amyloid plaque development, TREM2 upregulation accompanies a transcriptional shift in microglia from a homeostatic to an activated phenotype (22, 23), characterized by increased proportions of activated microglia with reduced process arborization but preserved cellular density (24). The functional consequences of TREM2 activation include promotion of amyloid plaque encapsulation and compaction, with TREM2 deficiency leading to more diffuse plaques containing elongated fibrils and increased surface area (25). The receptor’s interaction with APOE and APOE-containing lipoproteins represents a significant immune-related axis, suggesting ligand-specific mechanisms through which TREM2-APOE interactions may influence AD progression (5, 26). This relationship gains further support from observations that carriers of APOE and TREM2 risk variants exhibit reduced CD163+ amyloid-responsive microglia, implicating this subpopulation in disease susceptibility.

The protective potential of TREM2-mediated mechanisms is illustrated by studies of non-demented individuals with AD neuropathology (NDAN), where TREM2-enhanced microglial phagocytosis of damaged synapses may contribute to synaptic resilience (27). These protected synapses display distinct proteomic and miRNA signatures (28, 29), resist oligomeric Aβ and tau toxicity (30, 31), and associate with enhanced hippocampal neurogenesis (32) and preserved antioxidant capacity (33). Beyond central nervous system alterations, peripheral immune changes are evident in AD patients’ peripheral blood mononuclear cells (PBMCs), which demonstrate reduced TREM2+ monocytes, elevated plasma sTREM2, and impaired Aβ42 phagocytosis (34), indicating systemic dimensions of TREM2 dysregulation.

Emerging evidence has established TREM2 as a critical immune modulator in AD, influencing key pathological processes through microglia-dependent mechanisms. TREM2 attenuates tau hyperphosphorylation and neuronal apoptosis via activation of the PI3K/Akt/GSK-3β signaling pathway, both *in vitro* and *in vivo* (35). Specifically, TREM2 enhances phosphatidylinositol 3-kinase/protein kinase B (PI3K/Akt) activity, leading to inhibition of glycogen synthase kinase-3β (GSK-3β), which is a major driver of tau pathology in AD (36). Therapeutic strategies targeting this pathway may therefore ameliorate tau-related neurodegeneration, underscoring the therapeutic potential of TREM2 modulation (36), highlighting brain region-dependent roles of TREM2 in AD. Additionally, immunotherapeutic strategies designed to augment TREM2 signaling, such as CLEC7A-targeting antibodies that activate SYK, which have demonstrated efficacy in restoring microglial function in *TREM2*[*R47H*] mice (37). While the amyloid hypothesis remains influential (38), its limitations have become increasingly apparent, with TREM2-mediated neuroimmune dysfunction now recognized as a significant contributor to AD pathogenesis (39). The receptor’s essential role in supporting the disease-associated microglial signature (13, 18) and maintaining metabolic competence (8, 17) underscores the fundamental importance of microglial immune responses in AD progression.

## Immunological mechanisms of TREM2 signaling in AD

**Figure 2:**
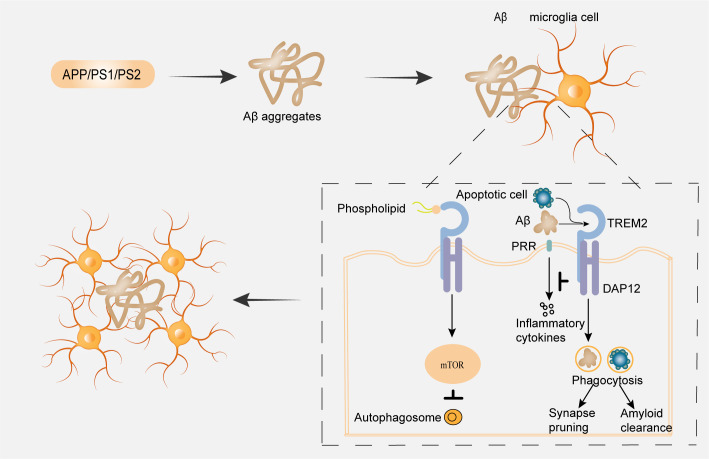
Neuroimmune crosstalk in Aβ pathology: microglial response to amyloid aggregation. Aβ aggregates, along with phospholipids released from apoptotic cells, are recognized by microglial receptors, including TREM2, which signals via the adaptor protein DAP12. Downstream signaling may promote the production of inflammatory cytokines and modulate mTOR activity, thereby influencing microglial phagocytosis and autophagic flux. These processes contribute to Aβ clearance; however, under chronic Aβ exposure, autophagy may become impaired, leading to dysregulated mTOR signaling, compromised Aβ clearance, and subsequent synaptic dysfunction.

TREM2, a key immunoreceptor in the TREM family, engages a variety of ligands, including Aβ and APOE to initiate intracellular signaling predominantly through the DAP12/DAP10 adaptor complex (14, 40), as illustrated in **Figure 2**. Its activity is modulated upstream by CD33, which also contributes to phagocytic and inflammatory processes (41). Ligand binding, particularly by Aβ, activates TREM2-mediated innate immune pathways, enhancing critical microglial functions such as phagocytosis, chemotaxis, and transcriptional reprogramming (42). As one of the most significant genetic risk factors in AD, TREM2 is itself regulated by Aβ and plays a vital role in controlling microglial density, synaptic phagocytosis, and neuronal transmission efficiency (40, 43). Furthermore, TREM2 mediates essential cross-talk between microglia and astrocytes during synaptic refinement (20), and collaborates with complement components C1q and C3 to eliminate superfluous synapses during circuit refinement (44). Beyond its role in pathogen clearance, TREM2 facilitates the non-inflammatory phagocytosis of apoptotic cells via recognition of aminophospholipids such as phosphatidylserine (45, 46), highlighting the functional significance of its ectodomain and soluble form (sTREM2).

Investigation into TREM2 variants has profoundly advanced our understanding of microglial pathobiology in AD. Loss of TREM2 function compromises the ability of microglia to encase Aβ plaques, proliferate locally, and adopt a DAM phenotype (47, 48), ultimately leading to severe neuritic dystrophy (25, 49). This is corroborated by findings in the *5XFAD* mouse model, where TREM2 deficiency curtails microglial proliferation and recruitment to plaques, thereby increasing amyloid pathogenicity (48). Consequently, TREM2-deficient mice develop larger, less compact plaques. Human carriers of the *TREM2 R47H* variant exhibit similar plaque morphology and neuritic dystrophy, confirming the loss-of-function characteristic of this mutation (25). Intriguingly, TREM2 deletion attenuates plaque load in early disease stages but exacerbates pathology later (50), supporting a model in which TREM2 enables microglia to surround and condense plaques, thereby limiting neuritic damage (49). This temporal disparity may be explained by impaired microglia-mediated Aβ seeding during early phases versus a loss of plaque-trimming capacity at advanced stages when compaction is critical (25, 49).

There are two theories to explain the mechanism of TREM2 impair the microglial response to amyloid pathology and exacerbate disease: 1) TREM2 signaling is necessary to reprogram cells from a homeostatic to a neuroprotective disease-associated phenotype (13), and 2) tonic TREM2 signaling is necessary to support microglial metabolism (8). Consistent with the metabolic hypothesis, TREM2-deficient microglia exhibit elevated autophagosomes, downregulation of biosynthetic and energy metabolism genes, and features of metabolic stress (8). Dietary administration of cyclocreatine ameliorated autophagic dysfunction, restored microglial clustering around plaques, and reduced neuritic dystrophy in TREM2-deficient mice (8). Transcriptomic profiling of plaque-associated microglia reveals enrichment of genes involved in lipid metabolism and phagocytosis (13, 14). However, it is crucial to contextualize the DAM phenotype within the broader spectrum of microglial heterogeneity (51). As highlighted in recent frameworks, ‘DAM’ describes a specific adaptation observed in particular contexts (e.g., around amyloid plaques in mouse models) and should not be viewed as a universal or monolithic endpoint. A more precise description involves defining microglial changes by their deviation from a homeostatic signature (e.g., downregulation of *P2RY12*, *CX3CR1*) rather than by a single label (13, 52). Beyond DAM, TREM2 is expressed in other context-dependent states, including senescent microglia (53). Crucially, TREM2 signaling is implicated in microglial senescence—a state characterized by a pro-inflammatory senescence-associated secretory phenotype (SASP). Evidence suggests that TREM2 deficiency reduces senescent microglia in AD models, indicating a functional dependency of this state on TREM2 (54). Furthermore, the TREM2-mTOR signaling axis may regulate metabolic pathways critical to cellular senescence (8). This creates a complex picture where TREM2 agonism could paradoxically influence both protective and detrimental inflammatory processes depending on the cellular context. This heterogeneity implies that the functional outcome of TREM2 signaling is critically shaped by the cellular and pathological context. Therefore, therapeutic TREM2 agonism may not simply ‘induce DAM’ but rather modulate the equilibrium across multiple microglial states. TREM2 also regulates APOE signaling, and intervention targeting the TREM2/APOE pathway restores microglial homeostasis and reduces neuronal loss in AD models (14).

Emerging evidence places TREM2 at the intersection of Aβ and tau pathology, where it restricts tau seeding and spreading from amyloid plaques (55). Integrated transcriptomic and functional studies using chimeric AD models reveal that TREM2 knockout impairs microglial survival, phagocytosis of APOE and other substrates, and SDF-1α/CXCR4-dependent chemotaxis, collectively dampening the amyloid response (56). Beyond proteinopathic mechanisms, TREM2 is also a central regulator of lipid metabolism both within the CNS and peripherally. In the brain, it influences cholesterol and myelin homeostasis (57, 58), binds to phospholipids, and affects the metabolism of some types of phospholipids (46, 59). In the periphery, TREM2 is associated with the occurrence and progression of obesity and its complications which are considered metabolic comorbidities of AD (60, 61). These observations collectively underscore that AD pathogenesis involves both direct Aβ toxicity and impaired TREM2-dependent immunomodulation.

## TREM2 variants and AD risk

The TREM2 receptor plays a fundamental role in orchestrating microglial immune responses, with its functional integrity being essential for maintaining neuroimmune homeostasis. Under physiological conditions, TREM2-mediated signaling supports microglial activation states necessary for effective tissue surveillance and response coordination. Microglial TREM2 serves as a central regulator in organizing responses to cerebral amyloid deposition, with plaque-associated microglia demonstrating coordinated upregulation of both TREM2 and its adaptor protein DAP12 in transgenic models (62). Beyond rare variants, genome-wide studies implicate TREM2 more broadly in AD risk (11, 12). TREM2 expression is also dysregulated in AD brains, with significant upregulation of both mRNA and protein in the frontal cortex of advanced cases relative to age-matched controls (63).

Alterations in TREM2 function, particularly through genetic variants, disrupt these homeostatic mechanisms and contribute to AD pathogenesis. To date, 63 coding variants in the TREM2 gene have been identified across diverse populations, with frequencies varying widely. Among these, the rare missense variant p. *R47H* (rs75932628) is consistently associated with a modest but significant increase in AD risk, as evidenced by multiple familial and case-control studies (64, 65). This variant was independently established as a risk factor in cohorts of European, North American (11), and Icelandic cohorts (12). Structurally, the *R47H* mutation localizes to TREM2’s immunoglobulin-like domain, disrupting ligand binding and impairing interactions with key ligands such as Aβ fibrils and phospholipids. This defect leads to deficient Aβ clearance, exacerbated plaque accumulation, and attenuated microglial activation (66). Postmortem studies of *R47H* carriers show reduced microglial clustering around plaques and compromised barrier function, further affirming the variant’s detrimental impact on microglial immune surveillance (67).

The functional consequences of TREM2 disruption extend beyond amyloid pathology. Cellular models provide mechanistic insight into TREM2 variant-driven pathogenicity. Co-culture of neurons with BV-2 microglia expressing *TREM2 R47H*, but not wild-type TREM2, resulted in significant neuronal loss, mediated by excessive phagocytosis of phosphatidylserine-exposing neurons. This gain-of-function phenotype suggests that the *R47H* variant heightens AD risk by potentiating synaptosis and neuronal engulfment, while wild-type TREM2 may exert a protective effect via cystatin F-dependent modulation of phagocytosis. These findings were corroborated across multiple microglial models, including BV-2, CHME-3, and iPSC-derived microglia, consistently showing that *R47H* enhances synaptic pruning and neuronal loss (68). Mounting evidence underscores the essential role of TREM2 in regulating microglial immune responses in AD. In mouse models, acquisition of the disease-associated microglia (DAM) phenotype is strictly TREM2-dependent (69). Transcriptomic analyses have not only confirmed the TREM2-dependence of DAM but also uncovered a novel oligodendrocyte response marked by *Serpina3n* and *C4b* expression, indicative of disrupted myelination and metabolic adaptation in neurodegeneration (69).

Clinically, TREM2 variant carriers exhibit phenotypes closely resembling sporadic AD across neuroimaging and neuropathological measures (11, 67). Among 563 AD patients in a clinic-based cohort, 12 carried p. *R47H* and presented with a significantly younger age at onset (70). Furthermore, p. *R47H* carriers from five late-onset AD families had a shorter disease duration compared to non-carriers (67). CSF biomarker analyses revealed elevated total tau and p-tau in variant carriers, despite Aβ42 levels commensurate with disease stage (71, 72). Elevated CSF sTREM2 levels were also observed (73), possibly reflecting compensatory neuroinflammatory mechanisms. The *TREM2 R47H* variant confers an approximately threefold increase in AD risk, substantial while less than that associated with APOE ϵ4, which elevates risk 3–4-fold in heterozygotes and 10–12-fold in homozygotes (74, 75).

Despite these robust genetic associations, the effect size of TREM2 variants remains insufficient for individualized risk prediction. Nevertheless, the conserved disruption of neuroimmune pathways in both TREM2-deficient models and human variant carriers solidifies TREM2’s role as a key immune modulator in AD pathogenesis (69). The functional interplay between TREM2 and microglial activation appears paramount for an effective response to Aβ plaques, with TREM2-dependent DAM differentiation representing a critical mechanism compromised in variant carriers. The concomitant emergence of reactive oligodendrocyte signatures in TREM2-deficient models points to broader neuroimmune disturbances affecting glial crosstalk and metabolic homeostasis (69), highlighting the complex interplay between genetic risk and neuroimmune dysfunction in Alzheimer’s disease.

## sTREM2 as a CSF biomarker in Alzheimer’s disease

Soluble TREM2 (sTREM2) is generated predominantly through proteolytic cleavage of membrane-bound TREM2 by ADAM10 and ADAM17, metalloproteases that shed its extracellular domain (76, 77). Alternatively, certain splice isoforms of TREM2 may be secreted independently of proteolysis (78). Importantly, sTREM2 is not an inert metabolite but a functionally active immune mediator. It participates in neuroimmune regulation by binding ligands and influencing microglial activation, contributing to the modulation of inflammatory responses within the central nervous system.

Elevated cerebrospinal fluid (CSF) levels of soluble TREM2 (sTREM2) show a strong correlation with tau pathology in AD, including total tau and phosphorylated tau, in contrast to the lack of association with Aβ42 (79, 80). This increase is detectable during preclinical stages and correlates with early neuronal injury (73, 79), reflecting its dynamic response to disease progression. The rise in CSF sTREM2 is widely interpreted as a proxy for microglial activation (79, 81), a hallmark of early neuroinflammatory processes in AD (82). This is corroborated by temporal patterns of sTREM2 increase following Aβ deposition and neuronal damage (83). However, its pathophysiological role is highly complex and exhibits clear stage-dependent dynamics across the Alzheimer’s disease continuum. Longitudinal cohort analyses reveal that higher or increasing levels of sTREM2 in the preclinical or mild cognitive impairment stages may be associated with a protective, compensatory microglial response, potentially attenuating amyloid and tau pathology accumulation and slowing neurodegeneration (84, 85). Conversely, in established AD dementia, specific patterns of sTREM2 elevation may reflect a transition to a chronic, dysfunctional neuroinflammatory state that is linked to more rapid structural and functional decline (86). This dichotomous role underscores that sTREM2 is not a unidimensional marker of neuroinflammation but a dynamic indicator whose clinical interpretation is contingent upon the existing pathological context (A/T/N status) and disease stage, reflecting the growing mechanistic clarity in the field (85, 87). Dysregulated TREM2 shedding may lead to sTREM2 overproduction, potentially impairing blood-brain barrier function and enhancing CSF influx (88, 89). This exchange is hypothesized to occur primarily at specialized brain barrier interfaces, particularly in the context of age- or disease-related dysfunction of the choroid plexus and the blood-brain barrier (BBB), rather than through non-specific vascular leakage (90). Alterations in these barrier systems could facilitate the entry of peripherally derived sTREM2 into the CSF compartment or influence the clearance of central sTREM2, thereby modulating its measurable levels and biological activity (88, 90).These characteristics position sTREM2 as a promising biomarker with substantial clinical utility. Its strong association with tau pathology supports its value in disease staging and differential diagnosis (91). CSF sTREM2 levels are further modulated by TREM2 coding variants, notably, carriers of the *R47H* mutation exhibit marked elevations. Thus, CSF sTREM2 represents a dynamic indicator of disease evolution, potentially enabling refined patient stratification and offering a surrogate endpoint in clinical trials targeting immune activation. However, the functional and diagnostic implications of elevated serum sTREM2 remain incompletely defined and warrant further mechanistic investigation (89), particularly regarding its role as a mediator of neuroimmune crosstalk in AD.

## TREM2 activated antibody and immunotherapy for Alzheimer’s disease

The development of TREM2-targeted immunotherapies represents a promising frontier in AD therapeutics, building upon the fundamental understanding of TREM2 signaling mechanisms. Upon ligand binding, the TREM2 ectodomain is proteolytically cleaved (76), enabling the intracellular fragment to associate with DAP12 and initiate downstream Syk-PI3K/MAPK signaling and IP_3_-mediated Ca²^+^ release, which collectively promote phagocytic activity (92, 93). Thus, TREM2 shedding is essential for microglial activation (94), with genetic studies further underscoring the role of TREM2 in regulating neuroinflammation in AD (95). The therapeutic potential of enhancing TREM2 signaling is further supported by experimental evidence demonstrating that crossing *5XFAD* mice with transgenic mice overexpressing human TREM2 reduces Aβ pathology (96). Additionally, myeloid cells with neuroprotective properties can be generated *in vitro* using TREM2 agonist antibodies (97).

Monoclonal antibodies represent a promising therapeutic avenue for Alzheimer’s disease (AD), leveraging their high specificity for targeted intervention. Preclinical and early-phase studies of TREM2-targeting antibodies, including AL002c, reported reduced amyloid pathology, attenuated neuroinflammation, and improved cognitive function in models. Mechanistically, these agents activate the DAP12/SYK pathway, stimulate microglial proliferation, and promote a disease-associated microglial (DAM) phenotype, favoring a modulated immune response (18, 98).

However, the subsequent Phase II INVOKE-2 trial yielded clinically neutral results. While AL002 successfully engaged its target, elevating soluble TREM2 (sTREM2) levels, it failed to significantly slow cognitive decline or reduce amyloid load in early AD patients. This outcome underscores a critical translational gap and highlights potential limitations such as suboptimal therapeutic timing, insufficient dosing or treatment duration, and the inherent challenge of modulating a pleiotropic immune receptor within a complex disease milieu (99). Furthermore, the occurrence of amyloid-related imaging abnormalities (ARIA) in this trial confirms that the potentiation of microglial activity carries inherent vascular risks, adding a critical safety dimension to the efficacy challenges observed(https://www.alzforum.org/news/conference-coverage/aria-inflammatory-reaction-vascular-amyloid).

Rather than invalidating TREM2 as a target, these findings emphasize the need for refined strategies. A mono−agent TREM2 approach may be insufficient to alter AD progression. Future directions should explore combination therapies—for example, with Aβ−clearing or tau−targeting agents—and next−generation candidates engineered for enhanced brain delivery (e.g., ATV: TREM2) or more precise patient stratification. Recent engineering advancements have further optimized TREM2-targeted immunotherapy. A high-affinity human TREM2-activating antibody was engineered as an antibody transport vehicle (ATV) by incorporating a monovalent transferrin receptor (TfR)-binding site to facilitate blood-brain barrier transcytosis. This innovative design demonstrates superior brain biodistribution and signaling potency compared to conventional anti-TREM2 antibodies following peripheral administration. In human iPSC-derived microglia, ATV: TREM2 induces proliferation and improved mitochondrial metabolism. Single-cell RNA sequencing and morphological analyses indicated that ATV: TREM2 promotes a metabolically responsive microglial state distinct from that induced by amyloid pathology alone. In an AD mouse model, ATV: TREM2 enhanced overall microglial activity and cerebral glucose metabolism, positioning ATV: TREM2 as a particularly innovative strategy for restoring microglial function in AD (100 ).Beyond direct TREM2 activation, alternative immunotherapeutic strategies have shown considerable promise. The monoclonal antibody Ab29 represents a novel approach by blocking interactions between LILRB2 and its ligands, thereby alleviating LILRB2-mediated inhibition of TREM2 signaling. This antibody enhances microglial phagocytosis, TREM2 signaling, migration, and cytokine responses to oligomeric Aβ-lipoprotein complexes in human microglia-like (hMGL) and human microglial cell clone 3 (HMC3) cell lines. *In vivo*, Ab29 treatment significantly increased microglial clustering around plaques and augmented amyloid phagocytosis in *5XFAD* mice (101). These results highlight LILRB2 antagonism as a novel therapeutic strategy for enhancing microglial function. Additionally, beyond antibody-based strategies, heat shock protein 60 (HSP60) emerging as a specific TREM2 agonist that binds to the receptor’s immunoglobulin-like domain, activating associated signaling pathways and enhancing microglial phagocytosis (102).

The collective evidence from these diverse approaches, such asAb29, HSP60, and ATV: TREM2, strongly indicates that TREM2-dependent microglial activation represents a viable therapeutic avenue that may delay AD onset and progression.

## Challenges and context-dependent complexities of TREM2 modulation

Emerging evidence, however, cautions against a unidirectional view of TREM2 agonism, revealing critical complexities that are pathology- and context-dependent. First, the impact of TREM2 signaling diverges fundamentally between parenchymal and vascular amyloid-β (Aβ) (103). Preclinical studies demonstrate that while loss of TREM2 exacerbates plaque deposition, it can significantly reduce cerebral amyloid angiopathy (CAA). In Tg-SwDI mice, TREM2 deficiency led to decreased CAA and perivascular microglial association despite an increased overall Aβ burden, indicating that TREM2 differentially regulates these two Aβ pools (104). Critically, neuropathological evidence from fatal ARIA cases demonstrates that anti-Aβ antibody therapy can trigger a robust perivascular inflammatory infiltrate, characterized by lymphocyte aggregation and activated reactive macrophages (103). This provides direct evidence that therapeutic intervention can markedly augment the immune cell response around vasculature, thereby driving severe inflammatory vasculopathy in susceptible individuals.

The clinical safety implications of this dichotomy are significant. Enhanced microglial phagocytic activity, induced by either anti-Aβ antibodies or TREM2 agonists, can precipitate amyloid-related imaging abnormalities (ARIA)—a vasculopathy associated with vascular amyloid (105). In severe instances, this may progress to cerebral amyloid-related inflammation, characterized by vasogenic edema, microhemorrhages, and, rarely, fatal arteritis, especially in APOE ϵ4 carriers with substantial CAA. This condition shares key features with CAA-related inflammation (CAA-ri), both involving perivascular microglial activation (106). Notably, ARIA occurrences in the Phase II INVOKE-2 trial of the TREM2 agonist AL002 confirm that this risk extends beyond anti-Aβ therapies(https://www.alzforum.org/news/conference-coverage/aria-inflammatory-reaction-vascular-amyloid).

Furthermore, the therapeutic outcome of TREM2 agonism is highly pathology-specific. In contrast to the context of amyloidosis, preclinical studies in models of demyelination (e.g., lysolecithin- or cuprizone-induced injury) have shown that TREM2 agonist antibodies can paradoxically impair injury resolution and limit myelin recovery (107). Therefore, therapeutic enhancement of TREM2 signaling, especially in patients with advanced vascular amyloid pathology, may unintentionally amplify detrimental neuroinflammatory responses around compromised vasculature. This highlights a pivotal safety consideration and argues that the net benefit of TREM2 agonism may inversely correlate with CAA burden.

## Therapeutic modulation of the TREM2 immune pathway

Neuroinflammatory processes in the central nervous system are orchestrated by multiple interconnected molecular pathways, with several key signaling molecules and receptors playing critical regulatory roles. Among these, spleen tyrosine kinase (SYK) has emerged as a central intracellular regulator of microglial function, mediating downstream signaling for immunoreceptors including TREM2, CD33, and CD22. SYK regulates diverse microglial processes including phagocytosis, Aβ compaction, and AKT/GSK3β signaling, highlighting its broad immunoregulatory functions in Alzheimer’s disease (108). Beyond its established role in antifungal immunity downstream of C-type lectin receptors (CLECs) (109), SYK activity is crucial for microglial facilitation of neurotoxic soluble Aβ oligomer consolidation into inert insoluble fibrils and dense plaques. Additional signaling mechanisms contribute to neuroinflammatory regulation, as evidenced by oxidative stress-induced calcium influx via the *TRPM2* channel activating cultured human microglia (57).

In AD, these neuroimmune pathways become dysregulated, contributing to sustained neuroinflammation and neurodegenerative processes. Proteomic studies have revealed that pathways driven by TNF-α, TGF-β, IL-1β, and TREM2 are critically involved in maintaining chronic neuroinflammatory states. The pathological significance of these pathways is substantiated by evidence that genetic ablation of *TRPM2* in *APP/PS1* mice mitigates Aβ-induced microglial activation, neuronal toxicity, and memory deficits (110), identifying *TRPM2* as a significant contributor to neuroinflammatory signaling in AD pathogenesis.

The growing understanding of these mechanisms has supported the development of therapeutic strategies targeting neuroimmune pathways. The pipeline of biomarker-informed therapeutic development now includes several promising candidates (**Table 1**). Monoclonal antibodies represent a major approach, with AL002 (a TREM2-activating antibody) and AL003 (an anti-CD33 antibody) directly targeting immunoreceptors involved in microglial regulation. Small molecule strategies include GC021109, a P2Y6 purinergic receptor agonist that enhances microglial phagocytosis and suppresses pro-inflammatory cytokine release, though it remains in early-phase trials (113). Similarly, the RAGE antagonist Azeliragon inhibits Aβ-RAGE interaction and has demonstrated neuroprotective effects in AD transgenic models (114). An emerging strategy involves selective inhibition of pro-inflammatory pathways without inducing broad immunosuppression. Targeting the NLRP3 inflammasome represents a promising approach in this regard. NLRP3 activation involves NF-κB-mediated transcriptional priming followed by inflammasome assembly and ASC speck formation, culminating in pyroptotic cell death. NLRP3 inhibitors have been shown to reduce amyloid burden and improve cognition in AD mouse models (115). These approaches, along with other investigational agents such as BAN2401 targeting Aβ protofibrils and next-generation non-steroidal anti-inflammatory drugs, collectively represent the diverse therapeutic strategies being evaluated in large-scale clinical trials for their potential disease-modifying effects in AD.

**Table 1:** Selected neuroimmune-targeted therapeutic candidates in development for Alzheimer’s disease.

Therapeutic candidate	Mechanism of action	Developmental stage	Reference
AL002	TREM2-activating monoclonal antibody	Phase 2 completed, did not meet primary endpoints; long-term extension discontinued	(99, 111)
AL003	Anti-CD33 monoclonal antibody	Phase 1 Terminated	(112)
GC021109	P2Y6 purinergic receptor agonist	Early-phase trials	(113)
ATV: TREM2	TREM2-activating monoclonal antibody engineered with a blood-brain barrier (BBB) transport vehicle (ATV) to enhance CNS delivery.	Preclinical	(100)
VHB937	TREM2-activating monoclonal antibody	Phase 1 (ongoing, NCT07094516)	ClinicalTrials.gov Identifier: NCT07094516
Azeliragon	RAGE antagonist	Preclinical efficacy in AD models	(114)
NLRP3 Inflammasome Inhibitors	Inhibits NLRP3 inflammasome activation	Preclinical efficacy in AD models	(115)
BAN2401	Monoclonal antibody targeting Aβ protofibrils	Large-scale clinical trials	(112)
Next-generation NSAIDs	Non-steroidal anti-inflammatory drugs	Large-scale clinical trials	(116)

## Discussion

This review establishes TREM2-mediated neuroimmunoregulation as a central pillar in Alzheimer’s disease (AD) pathogenesis. TREM2 functions as a critical modulator of microglia, operating at the nexus of Aβ aggregation, tau pathology, and chronic neuroinflammation. Human genetic studies, combined with mechanistic insights from experimental models, underscore a context-dependent dual role for TREM2, capable of either mitigating or accelerating disease progression depending on timing, genetic background, and the pathological milieu. The strong association between TREM2 variants—notably the *R47H* allele—and AD risk highlights the decisive influence of microglial immunity on disease trajectory. The TREM2-APOE axis exemplifies the intricate coupling of lipid metabolism and immune response, with APOE acting as a key TREM2 ligand that critically regulates microglial function. Furthermore, soluble TREM2 (sTREM2) has emerged as a dynamic biomarker of microglial activity; its correlation with tau, but not Aβ, pathology suggests it reflects ongoing neuroinflammation and neuronal injury beyond mere amyloid burden.

While recent reviews have authoritatively covered established aspects of TREM2 biology, this work provides a forward-looking synthesis by integrating several emerging frontiers. First, the prevailing CNS-centric view is expanded to a systemic immunometabolic framework. Growing evidence linking peripherally measured sTREM2 to conditions like obesity and type 2 diabetes positions AD within a broader dysregulated immunometabolic network, reframing its pathophysiology as a disorder of systemic immune homeostasis. Second, the critical dimension of microglial senescence is introduced. Beyond classical activation states, the dynamic crosstalk between senescent microglia and TREM2 signaling, particularly its potential influence on the senescence-associated secretory phenotype (SASP), illuminates a mechanistic bridge between fundamental aging processes, immune dysfunction, and sustained neuroinflammation. Third, a critical and balanced appraisal of translational efforts is presented. In contrast to predominantly optimistic narratives, recent neutral or negative outcomes are deliberately highlighted. The pivotal Phase II INVOKE-2 trial of the TREM2 agonist AL002, for instance, demonstrated clear target engagement (elevated sTREM2) but failed to slow cognitive decline or reduce amyloid burden, underscoring a critical disconnect between pharmacodynamic activity and clinical efficacy. This trial, along with evidence that TREM2 agonism can impair remyelination in preclinical demyelination models, reveals the profound context-dependence of therapeutic outcomes.

Collectively, these insights necessitate a paradigm shift from the simplistic goal of “activating TREM2” to the precise recalibration of microglial function within a specific pathological landscape. The recognition of profound microglial heterogeneity challenges the concept of promoting a uniform “neuroprotective DAM state.” Future strategies must account for this complexity through biomarkers capable of distinguishing microglial states (e.g., protective vs. senescent) and stringent patient stratification based on disease stage and co-pathologies. This is further emphasized by opposing evidence showing TREM2 modulation differentially affects parenchymal plaques and cerebral amyloid angiopathy (CAA), with the latter linked to the risk of amyloid-related imaging abnormalities (ARIA)—a safety signal also observed in the INVOKE-2 trial.

Consequently, future therapeutic success depends on contextually precise intervention. This mandates rigorous patient stratification using biomarkers of vascular amyloid, the identification of optimal therapeutic windows prior to the establishment of extensive vascular pathology or irreversible maladaptive microglial states, and the development of next-generation agents. Promising strategies include antibodies engineered for enhanced brain delivery (e.g., ATV: TREM2) and new candidates entering clinical evaluation (e.g., VHB937). Ultimately, translating the therapeutic promise of TREM2 will depend on achieving a balanced modulation that promotes parenchymal plaque clearance without inciting adverse cerebrovascular inflammation, embracing the pathway’s full complexity.
